# Regional variation in health care utilization in Sweden – the importance of demand-side factors

**DOI:** 10.1186/s12913-018-3210-y

**Published:** 2018-06-04

**Authors:** Naimi Johansson, Niklas Jakobsson, Mikael Svensson

**Affiliations:** 1 Health Metrics, Sahlgrenska Academy, University of Gothenburg, PO Box 463, SE-405 30 Gothenburg, Sweden; 2 Department of Economics, Karlstad University, SE-651 88 Karlstad, Sweden; 3 Norwegian Social Research (NOVA), Oslo, Norway; 4 Department of Economics, Williams College, Williamstown, MA USA

**Keywords:** Regional variation, Health care utilization, Demand, Panel data, Random effects

## Abstract

**Background:**

Differences in health care utilization across geographical areas are well documented within several countries. If the variation across areas cannot be explained by differences in medical need, it can be a sign of inefficiency or misallocation of public health care resources.

**Methods:**

In this observational, longitudinal panel study we use regional level data covering the 21 Swedish regions (county councils) over 13 years and a random effects model to assess to what degree regional variation in outpatient physician visits is explained by observed demand factors such as health, demography and socio-economic factors.

**Results:**

The results show that regional mortality, as a proxy for population health, and demography do not explain regional variation in visits to primary care physicians, but explain about 50% of regional variation in visits to outpatient specialists. Adjusting for socio-economic and basic supply-side factors explains 33% of the regional variation in primary physician visits, but adds nothing to explaining the variation in specialist visits.

**Conclusion:**

50–67% of regional variation remains unexplained by a large number of observable regional characteristics, indicating that omitted and possibly unobserved factors contribute substantially to the regional variation. We conclude that variations in health care utilization across regions is not very well explained by underlying medical need and demand, measured by mortality, demographic and socio-economic factors.

**Electronic supplementary material:**

The online version of this article (10.1186/s12913-018-3210-y) contains supplementary material, which is available to authorized users.

## Background

Levels of health care utilization and expenditure differ, not only among individuals but also across geographical areas within the same country [1, 2]. From a health policy perspective and concerning equity in care, it is fundamental to establish if the variations in health care utilization and expenditure across geographical entities are justified based on underlying demand factors, or if they are a sign of inefficiency, misallocation of public health care resources, or over- and underuse [3]. However, it is well documented that it is difficult to distinguish to what degree “demand” and “supply” of health care drives these regional variations due to the dependence between determinants [4, 5].

Factors known to determine health care utilization on an individual level include demand factors such as health status, demographic and socio-economic background [6]. Factors on the supply side that may drive regional variations in health care include access to care, medical practice and provider characteristics [1, 7]. If the population of an area has worse health status and a higher medical need, we may expect a higher level of health care utilization. On the other hand, if differences in medical practice and access to health care are the main drivers of regional variation, the variations are unwarranted and the health care system is not fulfilling the legislators’ intentions [3, 8, 9].

Despite a large number of studies demonstrating geographical variations in medical practice and health care, assessing what factors drive the variations are less studied [2, 3]. Cutler and Sheiner [7] used aggregated cross-sectional data of US Medicare expenditure and concluded that health status and demography accounted for 45% of the variation (measured using the standard deviation of residuals) in spending across hospital referral regions. Several of the more recent studies conclude that supply-side factors, such as physician preferences, style of practice and incentives, have more importance than the demand side in explaining geographical variation in health care [1, 4, 5, 10]. But, there are also other studies arguing that demand factors play a small but significant part in explaining regional variation [11], or even explain a large share of the variation [5, 12]. There are studies that also find that most [13], if not all [14], of regional variation in health care can be explained by observable characteristics of demographic, employment, health status and infrastructural factors.

In sum, we see somewhat conflicting empirical evidence on whether the demand side or the supply side is the driving force for regional variation in health care. This relates back to whether geographical differences can be justified based on underlying demand factors such as medical need or if they can be seen as a misallocation of common resources on the supply side. The purpose of this study is to examine the variations in outpatient health care utilization across the 21 Swedish regions. We explore to what extent regional variation of outpatient physician visits can be explained by common observable demand factors such as health status, demography and socio-economic structure. Similar to previous authors (e.g. [7, 13, 15]), we add covariates successively and estimate how much each set of covariates additionally explains the regional variation in physician visits, considering the estimated standard deviation of region-specific random intercepts. Furthermore, we evaluate how much of the regional variation remains unexplained after having adjusted for demand, and some basic supply-side factors.

Our study contributes to the literature by focusing on the demand side, in contrast to a large part of the previous literature where there has been a focus on supply-side factors (e.g. [4, 16–18]), probably in part due to difficulties to observe and measure factors on the demand-side [14]. Additionally, we contribute by assessing regional variation in health care in the level of utilization, as opposed to expenditure as in many previous studies have done (e.g. [4, 10–13]). This gives the advantage of not having to adjust for price and structural conditions associated with expenditure. Using visits to outpatient physicians as the outcome of interest have the advantage that it is a well-defined internationally comparable measure and that the vast majority of health care service takes place in outpatient care. Furthermore, it is purposeful to evaluate different types of health care separately as determinant factors are likely to differ [15, 19]. In the setting of a public, universal health care system, and using regional level data from national population and health care registers, we analyze regional variation in the general population. From an international perspective, the Swedish system is homogeneous with low levels of private insurance, few providers outside the public reimbursement system, and low patient cost sharing, which limits the effect of some potential confounders (price, diverse insurance terms, and access). The main reason to assess geographical variations on the aggregation level of regions, as opposed to for example municipal level or hospital level, is the decentralized structure of the Swedish health care system, described below, where the executive power lies with the regional governments.

In the universal health care system of Sweden, the central government sets principles and guidelines, whereas the responsibility to finance and provide health care lies with each of the 21 regions (county councils). Proportional taxes are set and collected within each region to fund the health care services. Providers are either public or private, but private providers are typically reimbursed through the public funds. Upon visits, patients are charged a (relatively small) copayment. Primary care physicians (general practitioners) are usually employed at a primary health care center, which is the first port of call for (non-acute) health related problems. Patients are assigned to a specific primary care center based on geographic proximity, but are free to register at another center of their choice. Specialists are usually employed at hospitals and may be involved in both out- and inpatient services. Supply is rationed by gate keeping and waiting times, and to allocate the patients’ priority the official rule is to follow the principle of need. Gate keeping is applied by the use of a phone triage system to primary care, and by the use of referrals to specialist care. In international comparisons, Swedish health care fares well on most health outcomes such as low levels of infant mortality and high life expectancy, but accessibility and long waiting times, which differ slightly across regions, are often-debated issues where the health care system comes out poorly.

## Methods

### Variables and data sources

The panel data set of the 21 Swedish regions (corresponding to Eurostat NUTS level 3 [20]) covers the period from 2001 to 2014. Regional borders have remained the same throughout this period, except a transfer in 2007 of one small municipality to the neighboring region, which is unlikely to affect our analysis. The data is gathered from online national registers of the Swedish municipality and region database, Statistics Sweden, the National Board of Health and Welfare and, the Swedish Association of Local Authorities and Regions. The data included are aggregated at the regional level from population registers (except unemployment, which is based on a sample survey) or health care registers, thus without any individual research subjects involved ethical review by the ethics committee was not necessary.

The focus of the analysis is on level of *utilization* of health care services (see e.g. [5, 15, 19]), rather than using *expenditure* as the outcome variable, as is the case in many other studies (see e.g. [4, 10, 12, 13]). Given the level of medical need and health care services used, regional variation in expenditure may arise due to regional labor market differences causing higher (lower) costs in some regions, therefore it makes sense to directly assess utilization. We assess two outcome variables: per capita visits to physicians in primary care (general practitioners) and per capita visits to specialists in outpatient care. The outcome variables are measured as the total number of physician visits occurred in the region, divided by the regional population. The purpose for separating visits to primary physicians and visits to specialists is because (i) previous authors have found determinants of variation and the degree of explanation to differ for different levels of health care [15, 19], and (ii) the development over time and variation across regions differ (see graphs in Additional files 1 and 2). Numbers include planned and non-planned visits, somatic and psychiatric care.

Determinants of health care use are grouped into four categories: mortality, demography, socio-economic structure, and supply. We use the regional mortality rate (standardized by age and gender of the average population) as a proxy for general level of health. The demographic covariates include proportion of women, proportion of seniors 65–79 years, proportion of seniors 80 years or older and, proportion of foreign born. To adjust for health (medical need), previous authors have used self- or physician assessed morbidity in combination with standardized mortality rate or demographic measures (see e.g. [5, 13, 15]). Mortality rate alone is a rather rough measure of health, but it is an objective, comparable measure based on high quality data of the whole population. Using health as a determinant of health care utilization assumes that health is exogenous, a simplifying assumption. Obviously, no claims of causal inference can be made but the aim of the analysis is to estimate the extent to which regional variation can be explained by included covariates.

The covariate set of socio-economic structure includes educational level, gross regional product (GRP) per capita, financial assistance and unemployment. These factors, are included because they are likely to affect individual health behavior and efficiency in health self-production, and thereby the motivation and capacity to seek health care. Covariates of supply include number of primary care centers (per 100,000 inhabitants), proportion of non-public providers and physicians (per 1000 inhabitants). Variables on the supply side are expected to affect the level of utilization as higher accessibility facilitates utilization, the possible presence of supplier induced demand and the interdependency between supply and other included explanatory covariates [19]. Also, for supply covariates, the issue of endogeneity is present as higher (lower) levels of utilization (due to need and/or demand) may increase (decrease) the number of providers, and we need to be careful not to interpret the relationship between supply and utilization as causal.

The patient out-of-pocket price is theoretically an important determinant of demand. In some recent Swedish studies it has been shown that out-of-pocket price has little effect on health care utilization, at least on an aggregated level [21, 22]. The out-of-pocket price of visits to primary physicians and to specialists are included in sensitivity checks.

Table 1 shows descriptive statistics for included variables. Due to restrictions of years covered for a few variables, the regressions of visits to primary physicians cover the period 2002–2014 (total 273 region-year observations) and the regressions of visits to specialists cover the period 2001–2013 (total 273 region-year observations). See Additional file 3 for a table specification of variable units, data sources and years covered.

**Table 1:** Descriptive statistics of included variables, from 2001 to 2014

	Mean	Min	Max	St.dev. Overall	St.dev. Between	St.dev. Within
Health care utilization						
Visits to primary physician (per capita)	1.37	0.99	2.02	0.17	0.16	0.08
Visits to specialist (per capita)	1.21	0.86	2.28	0.23	0.21	0.09
Mortality						
Mortality rate (per 100,000)	963.34	767.34	1155.01	77.94	48.59	61.80
Demography						
Women (%)	50.12	49.02	51.03	0.37	0.35	0.16
65–79 years (%)	13.74	9.58	17.93	1.73	1.31	1.16
80 years or older (%)	5.65	3.88	6.51	0.57	0.57	0.15
Foreign born (%)	10.86	3.88	23.43	4.12	3.89	1.59
Socio-economic structure						
Education lower (% with only primary educ.)	23.76	15.33	32.40	3.54	2.37	2.68
Education intermed. (% with secondary educ. at highest)	46.96	37.71	52.66	3.04	3.06	0.55
Education higher (% with some type of tertiary educ.)	27.75	19.60	43.48	4.81	4.33	2.29
GRP/capita (SEK 1000)^a^	328.92	241.79	571.00	52.76	48.59	22.96
Financial assistance (SEK 1000)^a^	1.02	0.45	1.82	0.27	0.24	0.13
Unemployment (%)	6.89	2.47	11.00	1.89	0.89	1.68
Supply						
Primary care centers (per 100,000)	13.14	7.81	22.76	3.05	2.92	1.06
Non-public primary care (%)	25.31	0.00	67.31	17.03	14.65	9.21
Density of physicians (per 1000)	2.95	2.21	4.27	0.41	0.33	0.24
Copayments						
Copayment primary (SEK)^a^	147.01	99.77	209.69	26.03	18.90	18.33
Copayment specialist (SEK)^a^	284.15	199.64	350.00	36.41	25.62	26.42

^a^Prices in 2014 price level, 1 SEK ≈ €0.10

#### Description of health care utilization in Sweden

**Fig. 1:**
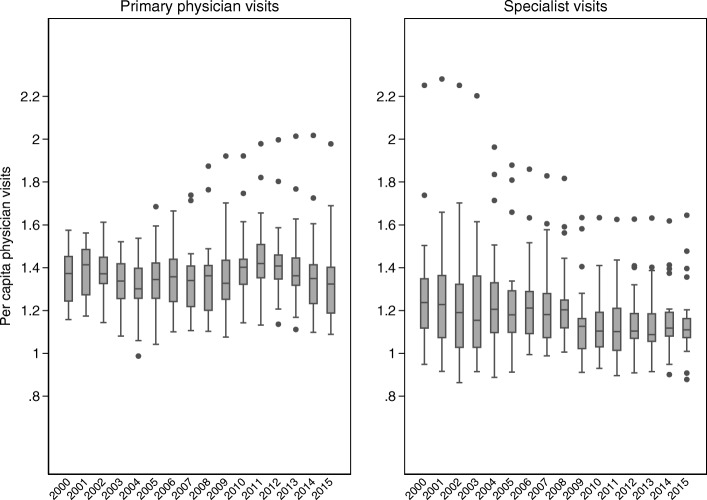
Spread of region per capita number of visits to physician in primary care and to specialist. Note. Outliers defined as observations more than 1.5*(inner quartile range) away from the 25th resp. 75th percentile

In terms of physician visits per capita, health care utilization in Sweden is low in international comparison. In 2014, the per capita number of physician’s consultations was e.g. 4.3 in Norway, 6.3 in France, 7.6 in Canada (2013), whereas it was only 2.9 in Sweden [23]. Separating visits into primary and specialist care, the national per capita has fluctuated around 1.4 throughout the 2000s for both levels of physician’s care (see graph in Additional file 1). Across the 21 regions of Sweden, the number of visits to physicians in primary care ranged from 1.2 to 1.6 per capita in 2000, and from 1.1 to 2.0 per capita in 2015. As shown in the left panel of Fig. 1, there are only a few observations (actually two regions, the capital region of Stockholm and a small southern region, Halland) that have considerably higher values during the second part of the period. Median, inner quartile range and min–max range of the box plots vary over the years, but not in an increasing or decreasing manner. See graph in Additional file 2 for a display of the development over time for each region separately, where no obvious geographical patterns over time are seen.

For visits to outpatient specialists, the region averages ranged from 0.9 to 2.2 in 2000 and from 0.9 to 1.6 in 2015. The right panel of Fig. 1 show that the variation between regions has decreased over the period; both the inner quartile range and the min–max range seem to diminish over the years. There are more outliers among specialist visits than among visits to primary physicians; again, the capital region of Stockholm has the highest values almost every year, but two large urban regions with major university hospitals (Uppsala and Skåne) also stand out.

**Fig. 2:**
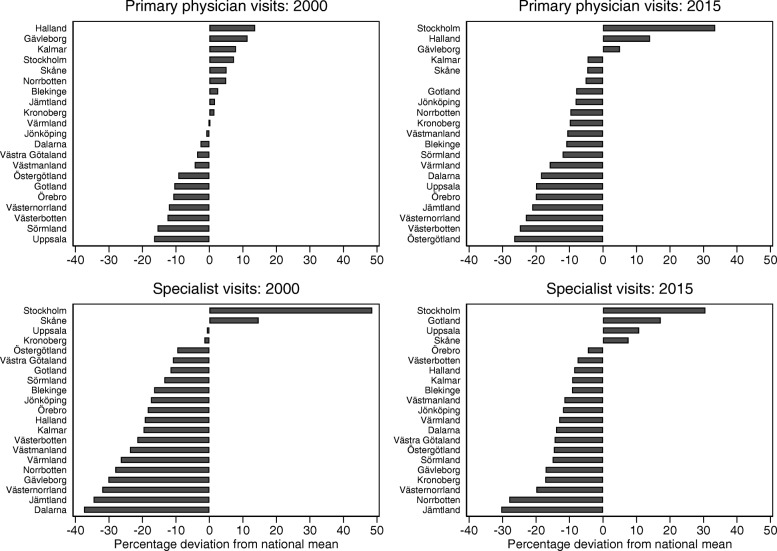
Relative differences (percentage deviation from national mean) between region means of visits to physicians in primary care and visits to specialists respectively. Note. Zero represents the national mean number of visits, and the scale is the percentage deviation from the national mean. The national mean is heavily dependent on Stockholm’s figures as Stockholm holds about 2 million (20%) of Sweden’s population

Displaying only two years from the panel separately gives the advantage of marking out each region, enabling a comparison of regional ranking over the years. Figure 2 (top panels), shows how the variation between regions in visits to primary physicians is greater in 2015 than in 2000. In 2000, the relative difference across the regions ranged from around ±15% of the national mean, whereas in 2015 the range had increased to about ±30% of the national average. For specialist visits in outpatient care (lower panels in Fig. 2), the relative difference across the regions in 2000 ranged from 35% below to 50% above the national mean, but in 2015 the range was ±30% of the national average. In general, low-utilizing regions remain at the bottom and high-utilizing regions remain at the top, although there are some regions that have changed considerably over this time period.

#### Empirical approach

We apply a random effects model, estimated by GLS, with model specification

1 $$ {y}_{it}=\alpha +{\boldsymbol{\beta} \boldsymbol{X}}_{\boldsymbol{it}}+{\delta}_i+{\varepsilon}_{it} $$

where $$ {\delta}_i\sim N\left(0,{\sigma}_{\delta}^2\right),{\varepsilon}_{it}\sim N\left(0,{\sigma}_{\varepsilon}^2\right) $$.

*y*_*it*_ is the outcome variable, per capita visits to physician, in region *i* in year *t*. ***X***_***it***_ is a vector of explanatory variables grouped in the four covariate sets as shown in Table 1 (mortality, demography, socio-economics, and supply). *δ*_*i*_ is a random effect of unobserved, time-invariant disturbance, allowing for region-specific intercepts, and *ε*_*it*_ is an error term [24]. The random effects, *δ*_*i*_, is assumed to be normally distributed with mean zero and variance ${\sigma}_{\delta}^2$ [25]. The variance is estimated by ${\widehat{\sigma}}_{\delta}^2$, yielding a measure of the (unexplained) regional variation. Due to a limited data set of 21 regions, we use a stepwise backward elimination of covariates with *p*-values > 0.2 [26]. We estimate two models, Model 1 with visits to physicians in primary care as the dependent variable; and Model 2 with visits to specialists in outpatient care as the dependent. The two models are estimates five times each, adding covariate sets successively. By looking at how the standard deviation of the random effects, ${\widehat{\sigma}}_{\delta }$, is reduced in successive regressions, we can assess the amount of regional variation explained. The statistical analysis is performed using Stata software 14.1.

## Results

Assessing the stepwise backward elimination of covariates, we include eight covariates in Model 1 and nine covariates in Model 2. Mortality rate, as a proxy for the general level of health, is kept in both models despite being non-significant. In Model 1, of visits to physician in primary care, the mortality rate reduce ${\widehat{\sigma}}_{\delta }$ only by 1%, and adding demographic covariates actually introduces more variation (Table 2, for full regression results see tables in Additional files 4 and 5). Including covariates of socio-economics, 11% of regional variation is explained, and including supply the added covariates together explain 33% of variation on regional level, leaving two thirds of regional variation unexplained.

**Table 2:** Estimated regional variation and degree of explanation

	Model 1: Visits to primary physicians		Model 2: Visits to specialists	
	${\widehat{\sigma}}_{\delta }$	% of ${\widehat{\sigma}}_{\delta }$ explained	${\widehat{\sigma}}_{\delta }$	% of ${\widehat{\sigma}}_{\delta }$ explained
Unadjusted	0.1597	–	0.2152	–
Adjusted for:				
Mortality	0.1580	1.1%	0.1761	18.2%
+Demography	0.1652	−3.4%	0.1086	49.5%
+Socio-economy	0.1418	11.2%	0.1025	52.4%
+ Supply	0.1064	33.4%	0.1069	50.3%

${\widehat{\sigma}}_{\delta }-$Estimated standard deviation of random effect *δ*_*i*_ (variation on regional level)

% of ${\widehat{\sigma}}_{\delta }$ – percentage of regional variation explained by included covariates

In Model 2, of visits to specialists, the estimated regional variation is larger than that of visits to primary physicians (Table 2). The mortality rate reduce ${\widehat{\sigma}}_{\delta }$ by 18%, and adding demographic covariates explain another 31% of the regional variation. Including socio-economic and supply covariates marginally adds to the degree of explanation, and in total included covariates account for 50% of the regional variation. In both Model 1 and 2, about 70% of the total variation after adjusting for covariates is found at the regional level, the remaining 30% being variation over time (Additional files 4 and 5).

In robustness checks, the results do not change from including neither year-dummies nor cluster robust standard errors. Excluding Stockholm County Council from the analysis, the unadjusted variation in smaller and less of regional variation is explained by included covariates (19% and 43% in Model 1 and 2 respectively). Including patient out-of-pocket price introduces more regional variation in Model 1 but explains additionally 5% of regional variation in Model 2. (Results from robustness checks are available on request).

## Discussion

Regional differences in health care utilization in Sweden are, as in many other countries, of relatively large magnitude. If the capital region of Stockholm had had the same per capita number of visits as the lowest utilizing regions in 2015, it would correspond to 2 million visits less in Stockholm primary care, and 1.5 million visits less in specialist care (now a total of 4.4 and 3.5 million visits, respectively). Even after adjusting for regional mortality, demography, socio-economy and supply, at least 50% of regional differences remain.

Our results for visits to specialists are in line with previous findings indicating that demography and health explain 45–55% of regional variations in health care utilization [5, 19] and in health care expenditure [7, 13], while our results for visits to primary physician deviate from these. The different measures of outcome (type of expenditure or utilization) make direct comparison to some of these studies difficult. In contrast to our findings, Kopetsch and Schmitz [19] find socio-economic structures and supply additionally explain regional variation in specialist visits. And for visits to primary physician, Kopetsch and Schmitz [19] find demography and health explain a large portion of the regional variation, while we find demography and mortality are irrelevant for variation in primary care.

Our study differs from Kopetsch and Schmitz [19] in several aspects (setting, type of data set, size of area considered, measure of regional variation) which might explain the deviating results. It is also important to acknowledge the difference in using only mortality as our measure of health status, while Kopetsch and Schmitz [19] include a composed measure of morbidity, see further discussions below. Our findings that mortality and demography explain a larger share in specialist visits compared to primary care might be related to the institutional settings of gate keeping and waiting times, which is applied stricter in specialist care. And our findings that supply-side factors, measured as the per capita number of providers and physicians, add to the degree of explanation in primary care but not in specialist care, is probably primarily related to accessibility, as more choices usually are available in primary care.

Our findings show that 50–67% of regional variation in physician visits remains unexplained by included covariates and imply that we cannot rule out that at least part of the variations may be related to misallocation of resources or inefficiencies. But for visits to specialist we show that since 50% of variations are explained by mortality and demography at least half of the variations are not due to inefficiencies. We cannot make the same claim for primary physician visits since mortality and demography do not explain the regional variations.

The large proportion of variation remaining unexplained also suggest that other omitted factors as well as measurement errors in included covariates may contribute to the variation. Potentially important determinants are for example cultural context (including social norms of health care seeking behavior), rurality, and supply-side specific institutional settings that differ across regions. It is possible that patients and physician in a region develop a regional culture, potentially due to system incentives. Stiernstedt [27] pointed out that the deviating reimbursement scheme of Stockholm County Council may have had a major influence on the large increase in primary physician visits in Stockholm seen from 2007 and onward. In Stockholm, a larger proportion of the reimbursement to primary care providers was attributed to the number of visits and a smaller proportion to the number of patients listed, in comparison to other regions’ reimbursement schemes [28].

The deviating reimbursement scheme in Stockholm, with some incentives for providers to encourage additional visits, may contribute to the presence of supplier induced demand, which traditionally has been seen as a theoretical cause of regional variation in health care [1]. But, it is very difficult to causally show the existence of supplier induced demand without details on e.g. patient preferences. With present study design, we cannot confirm the existence of supplier induced demand, and we cannot rule it out either. Cutler et al. [4] showed while organizational factors matter, physician treatment preferences are more important for regional variation in health care expenditure.

The outcome measures are based on physician visits made in each region, irrespective of patient’s residence. This is a limitation in our analysis as there may be spillover effects of people consuming health care outside their home-region. The main reason for patients to consume care outside their home-region is unplanned primary or acute care, for example due to a sudden illness or an accident [29]. In 2016, the ratio of specialist visits consumed by region-inhabitants to visits produced in the region, varied between 0.3–3.6 across the regions [30]. However, this does not seem to be associated with overall regional variations in outpatient specialized care when controlling for other covariates (results available on request). A previous study modeled spatial effects between the 402 German counties and found spillover effects to be negligible [13]. It would have been of interest to take into account and measure the variations within regions, such as nesting on municipal level and nesting on hospital and primary care center level. Due to limitations in data availability we were not able to do this type of analysis.

Using the mortality rate along with a set of demographic covariates to account for the regional level of health and medical need has its limitations, and can be seen as a measurement error. The additional inclusion of some measure of morbidity, such as self- or physician-assessed health status from surveys or insurance records, may capture the general health status better. The disadvantage of self- or physician-assessed health status is the risk of selection bias, as those visiting a physician are more likely to be diagnosed and to be aware of their underlying health status. The mortality rate alone is in a sense a crude measure of health, but it is an objective, comparable measure, and in our case based on quality register data of the whole population.

In a random effects model we assume independence between included covariates and the random intercepts, just as the error term is assumed to be independent [24]. This is a limitation in our choice of model, but given the need of a model that deal with dependence between observations and that can estimate variation on different levels, the random effects model is our preferred choice. A fixed effects model is not suitable for our cause since it cannot measure variation between groups (regions), and it assesses only the within variation, eliminating all region-specific time-invariant factors. Using an aggregated panel data set, we can assess the variation on regional level and over time, but cannot say anything about the variation on individual level. Our model cannot answer the (normative) question of what factors are justified determinants of regional variation. Medical need, as an acceptable reason for diverse health care use, is not easily measured objectively. From the set of covariates included here, mortality rate along with some demographic and socio-economic factors might together capture medical need.

In general, coefficient estimates show expected signs, for example, a higher education level is associated with a lower level of visits in primary care, but a higher level of visits in specialist care. But our results show that a larger proportion of 65–79 year olds is associated with lower levels of visits to the specialist. Both of these issues might be related to the location of universities and university hospitals, something we have not adjusted for. This shows that even though demography explain a good share of the regional variation in specialist visits, it is not necessarily the expected association (e.g. that an older population means more health care used). An unexpected finding is that regional variation does not always decrease when adding a set of covariates, true for demography in primary visits and for supply in specialist visits. With a closer look in the regression results, the proportion of 65–79 year olds have a positive association with the level of primary visits, but only variation over time is reduced in this step. This indicates that the effect within a region over time and the effect between regions are not equal, which is an assumption in the random effects model [25]. Despite a significant association between a given independent variable and the dependent variable, the degree of explanation can differ on different hierarchical levels (across regions and over time).

Potential substitution between outpatient and inpatient services is not taken into account in our analysis, so we can neither reject nor confirm that substitution plays a role. Running Pearson’s correlation between primary physician visits and average number of hospital days, and between specialist visits and hospital days in our data set, we find a low, negative correlation, − 0.11 and − 0.23 respectively. Looking at some previous evidence on the correlation between different type of health care services use, Welch et al. [31] found a positive correlation between outpatient and inpatient services in US Medicare spending. Another example is Zhang et al. [32] who found that health-adjusted pharmaceutical spending in US Medicare does not correlate with nondrug medical spending, indicating that drugs are a substitute for some patients and a complement for others. Potential substitution between outpatient primary and specialist care was considered in some of our earlier tests, i.e. including specialist visits as an independent variable in the regression of primary physician visits and vice versa. However, being insignificant they were dropped out without affecting the degree of explanation.

## Conclusions

In summary, we use an observational, longitudinal study design with the 21 Swedish regions over 13 years and run a random effects model dealing with dependence across regions using region specific random intercepts. Our results show that regional mortality and demography do not explain variation in visits to primary physicians, while these factors explain a large portion of the variation in visits to specialists. Having adjusted for mortality and demography, the regional level of socio-economy and especially supply does not add to the degree of explanation of variation in specialist visits but accounts for a large portion of variation in primary physician visits, indicating that access to care matters for utilization of primary care. Overall, 70% of the (unexplained) variation in the sample is variation across regions, the remaining being variation over time. We conclude that regional variations in health care utilization cannot be solely explained by underlying medical need and demand, and that this warrant further research and analyses to form policy proposals.

## Additional files

Additional file 1: Figure of the national mean per capita number of visits to physicians from 2000 to 2015, primary and specialist outpatient care respectively. (PDF 56 kb)

Additional file 2: Figure of a full overview of per capita number of physician visits to primary care and specialists in all 21 regions (county councils) from 2000 to 2015. (PDF 64 kb)

Additional file 3: **Table S1.** Specification of variables and data sources. (DOCX 18 kb)

Additional file 4: **Table S2.** Regression results for Model 1, dependent variable visits to physician in primary care. (DOCX 20 kb)

Additional file 5: **Table S3.** Regression results for Model 2, dependent variable visits to specialist. (DOCX 20 kb)

## Abbreviations

GRP: Gross regional product
SEK: Swedish kronor
