# Supplementary figures and images for: Regional variation in health care utilization in Sweden – the importance of demand-side factors
Source: BMC Health Serv Res. 2018 Jun 4;18:403. doi: 10.1186/s12913-018-3210-y (PMC5987462; doi:10.1186/s12913-018-3210-y)

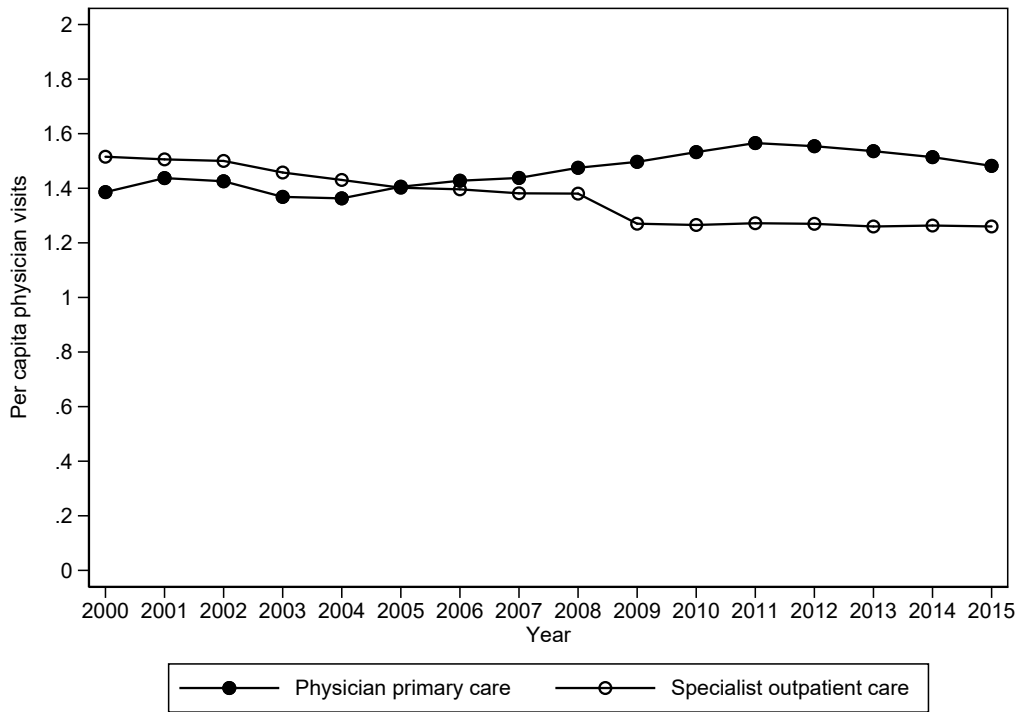

Supplement: Supplementary file 1 — Figure of the national mean per capita number of visits to physicians from 2000 to 2015, primary and specialist outpatient care respectively. (PDF 56 kb) [file 12913_2018_3210_MOESM1_ESM.pdf]

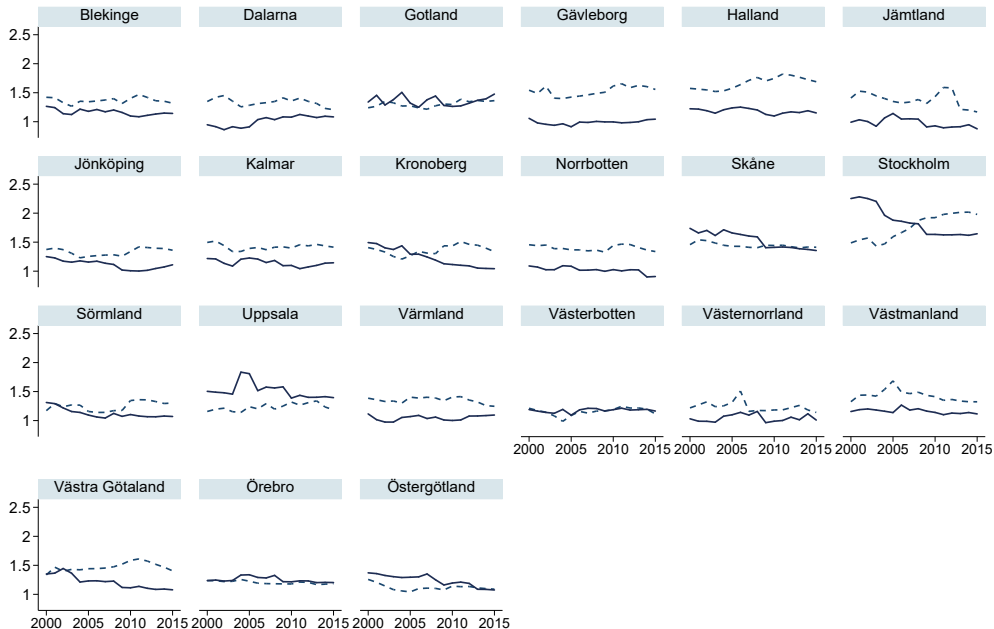

----- Physician primary care

————— Specialist outpatient care

Supplement: Supplementary file 2 — Figure of a full overview of per capita number of physician visits to primary care and specialists in all 21 regions (county councils) from 2000 to 2015. (PDF 64 kb) [file 12913_2018_3210_MOESM2_ESM.pdf]
