# Supplementary material for: Regional variation in health care utilization in Sweden – the importance of demand-side factors
Source: BMC Health Serv Res. 2018 Jun 4;18:403. doi: 10.1186/s12913-018-3210-y (PMC5987462; doi:10.1186/s12913-018-3210-y)
Supplement: Supplementary file 3 — Table S1. Specification of variables and data sources. (DOCX 18 kb) [file 12913_2018_3210_MOESM3_ESM.docx]

**Additional file 3**

**Table A1** Specification of variables and data sources

|  |  | Specification | Data source | |
| --- | --- | --- | --- | --- |
| Dependent variables | | | |  |
|  | Visits to physician primary (2002–2014) | Visits to primary care physicians (GP), average number per capita. | Swedish municipality and region database (Kolada) | |
|  | Visits to specialist  (2001–2013) | Visits to outpatient specialists (somatic or psychiatric), average number per capita. | Swedish municipality and region database (Kolada) | |
| Mortality | | | |  |
|  | Mortality rate | Number of persons deceased, standardized by average population in 2000 | National Board of Health and Welfare (Socialstyrelsen) | |
| Demography | | | |  |
|  | Women | Percentage of population being female | Statistics Sweden (SCB) | |
|  | Seniors (65–79) | Percentage of population 65–79 years. | Statistics Sweden (SCB) | |
|  | Seniors (80 or older) | Percentage of population 80 years or older. | Statistics Sweden (SCB) | |
|  | Foreign born | Percentage of population born abroad. | Statistics Sweden (SCB) | |
| Social capital & economic structure | | | |  |
|  | Education primary | Percentage of population with only primary education, (9 years). | Statistics Sweden (SCB) | |
|  | Education secondary | Percentage of population with secondary education (as their highest) | Statistics Sweden (SCB) | |
|  | Education higher | Percentage of population with some type of tertiary education. (Incl. approx. 1–3 % with unknown education). | Statistics Sweden (SCB) | |
|  | GRP/capita^a^ | Gross regional product, average per capita, thousand SEK. | Statistics Sweden (SCB) | |
|  | Financial assistance^a^ | Transfer of financial assistance from social services, average thousand SEK per capita. | National Board of Health and Welfare (Socialstyrelsen) | |
|  | Unemployment | Percentage. (Break in time series: 2000–2004 16–64 years, 2005–2015 15–74 years. Survey based.) | Statistics Sweden (SCB) | |
| Supply | | | |  |
|  | Primary care centers | Number of primary care centers per 100,000 inhabitants. | Swedish Association of Local Authorities and Regions (SKL) | |
|  | Non-public primary care | Percentage of primary care centers run by non-public provider. | Swedish Association of Local Authorities and Regions (SKL) | |
|  | Density of physicians | Number of physicians employed in health care (public and private), per 1,000 inhabitants. | National Board of Health and Welfare (Socialstyrelsen) | |
| Copayments | | | |  |
|  | Copayment physician primary care^a^ | Copayment for a visit to primary care physician. SEK. | Swedish Association of Local Authorities and Regions (SKL) | |
|  | Copayment specialist^a^ | Copayment for a visit to specialist (outpatient). SEK. | Swedish Association of Local Authorities and Regions (SKL) | |

1. Prices at 2014 price level, 1 SEK ≈ € 0.10
