# Supplementary material for: Regional variation in health care utilization in Sweden – the importance of demand-side factors
Source: BMC Health Serv Res. 2018 Jun 4;18:403. doi: 10.1186/s12913-018-3210-y (PMC5987462; doi:10.1186/s12913-018-3210-y)
Supplement: Supplementary file 4 — Table S2. Regression results for Model 1, dependent variable visits to physician in primary care. (DOCX 20 kb) [file 12913_2018_3210_MOESM4_ESM.docx]

**Additional file 4**

**Table A2** Regression results for Model 1, dependent variable visits to physician in primary care

|  | | + Mortality | | + Demo-graphy | + Socio-economy | + Supply |  | Only significant covariates |
| --- | --- | --- | --- | --- | --- | --- | --- | --- |
| Mortality | |  | |  |  |  |  |  |
|  | Mortality rate | -0.0004*** (0.0001) | | -0.0002 (0.0001) | 0.0002 (0.0002) | 0.0001 (0.0002) |  |  |
| Demography | |  | |  |  |  |  |  |
|  | 65–79 years |  | | 0.014** (0.007) | 0.051*** (0.012) | 0.041** (0.011) |  | 0.039*** (0.011) |
|  | 80+ years |  | | -0.083*** (0.032) | 0.012 (0.037) | -0.026 (0.035) |  |  |
| Socio-economy | |  | |  |  |  |  |  |
|  | Education secondary |  | |  | -0.057*** (0.012) | -0.046*** (0.011) |  | -0.050*** (0.010) |
|  | Education higher |  | |  | -0.009 (0.007) | -0.013** (0.006) |  | -0.015*** (0.005) |
|  | GRP/capita |  | |  | 0.0008** (0.0003) | 0.001*** (0.0003) |  | 0.001*** (0.0003) |
| Supply | |  | |  |  |  |  |  |
|  | Primary care centers |  | |  |  | 0.019*** (0.005) |  | 0.019*** (0.004) |
|  | Non-public primary care |  | |  |  | 0.001* (0.001) |  | 0.001*  (0.001) |
|  | |  | |  |  |  |  |  |
| Constant | | 1.726*** (0.089) | | 1.844*** (0.293) | 3.088*** (0.623) | 2.671*** (0.563) |  | 2.967*** (0.447) |
| ${\hat{\boldsymbol{\sigma}}}_{\boldsymbol{\delta}}$ | | 0.1580 | | 0.1652 | 0.1418 | 0.1064 |  | 0.1021 |
| ${\hat{\boldsymbol{\sigma}}}_{\boldsymbol{\varepsilon}}$ | | 0.0823 | | 0.0810 | 0.0756 | 0.0716 |  | 0.0715 |
| % of variation on regional level | | 0.7864 | | 0.8061 | 0.7786 | 0.6879 |  | 0.6710 |
| R^2^ Within | | 0.0654 | | 0.1014 | 0.2155 | 0.2942 |  | 0.2934 |
| Between | | 0.0710 | | 0.0637 | 0.1547 | 0.3886 |  | 0.3529 |
| Overall | | 0.0585 | | 0.0715 | 0.1604 | 0.3686 |  | 0.3399 |
|  | |  |  |  |  |  |  |  |
| Observations | | 273 | | 273 | 273 | 273 |  | 273 |
| Number of regions | | | 21 | 21 | 21 | 21 |  | 21 |

Notes. Years included 2001–2013. ***, **, * correspond to statistical significance at 1 %, 5 % and 10 % level respectively.
