# Supplementary material for: Regional variation in health care utilization in Sweden – the importance of demand-side factors
Source: BMC Health Serv Res. 2018 Jun 4;18:403. doi: 10.1186/s12913-018-3210-y (PMC5987462; doi:10.1186/s12913-018-3210-y)
Supplement: Supplementary file 5 — Table S3. Regression results for Model 2, dependent variable visits to specialist. (DOCX 20 kb) [file 12913_2018_3210_MOESM5_ESM.docx]

**Additional file 5**

**Table A3** Regression results for Model 2, dependent variable visits to specialist

|  | | + Mortality | + Demo-graphy | + Socio-economy | + Supply |  | Only significant covariates |
| --- | --- | --- | --- | --- | --- | --- | --- |
| Mortality | |  |  |  |  |  |  |
|  | Mortality rate | 0.0006  (0.0001) | 0.0001 (0.0001) | 0.0002 (0.0002) | 0.0002 (0.0002) |  |  |
| Demography | |  |  |  |  |  |  |
|  | Women |  | 0.363*** (0.051) | 0.336*** (0.052) | 0.319*** (0.053) |  | 0.320*** (0.052) |
|  | 65–79 years |  | -0.005 (0.008) | -0.045*** (0.011) | -0.047*** (0.011) |  | -0.048*** (0.011) |
|  | 80+ years |  | 0.014  (0.031) | 0.013  (0.036) | 0.010  (0.036) |  |  |
| Socio-economy | |  |  |  |  |  |  |
|  | Education secondary |  |  | 0.043*** (0.011) | 0.041*** (0.011) |  | 0.040*** (0.010) |
|  | Education higher |  |  | 0.027*** (0.006) | 0.015* (0.008) |  | 0.013*  (0.008) |
|  | Financial assistance |  |  | 0.121*** (0.042) | 0.104** (0.043) |  | 0.103**  (0.042) |
|  | Unemployment |  |  | -0.016*** (0.005) | -0.015*** (0.005) |  | -0.016*** (0.004) |
| Supply | |  |  |  |  |  |  |
|  | Physician density |  |  |  | 0.087** (0.042) |  | 0.082**  (0.042) |
|  | |  |  |  |  |  |  |
| Constant | | 0.610*** (0.100) | -16.876*** (2.609) | -18.042*** (2.709) | -17.036*** (2.765) |  | -16.718*** (2.724) |
| ${\hat{\boldsymbol{\sigma}}}_{\boldsymbol{\delta}}$ | | 0.1761 | 0.1086 | 0.1025 | 0.1069 |  | 0.1026 |
| ${\hat{\boldsymbol{\sigma}}}_{\boldsymbol{\varepsilon}}$ | | 0.0875 | 0.0767 | 0.0697 | 0.0693 |  | 0.0697 |
| % of variation on regional level | | 0.8020 | 0.6673 | 0.6842 | 0.7039 |  | 0.6840 |
| R^2^ Within | | 0.1677 | 0.2952 | 0.3673 | 0.3875 |  | 0.3798 |
| Between | | 0.3619 | 0.3776 | 0.4997 | 0.4497 |  | 0.4788 |
| Overall | | 0.0520 | 0.3645 | 0.4787 | 0.4396 |  | 0.4630 |
|  |  |  |  |  |  |  |  |
| Observations | | 273 | 273 | 273 | 273 |  | 273 |
| Number of regions | | 21 | 21 | 21 | 21 |  | 21 |

Notes. Years included 2002–2014. ***, **, * correspond to statistical significance at 1 %, 5 % and 10 % level respectively.
